# Does Early Career Achievement Lead to Earlier Death? Assessment of the Precocity-Longevity Effect in Professional Basketball Players

**DOI:** 10.3389/fpubh.2016.00258

**Published:** 2016-11-16

**Authors:** Nick Wattie, Srdjan Lemez, Chris I. Ardern, Michael Rotondi, Joseph Baker

**Affiliations:** ^1^Faculty of Health Sciences, University of Ontario Institute of Technology, Oshawa, ON, Canada; ^2^School of Kinesiology and Health Science, York University, Toronto, ON, Canada

**Keywords:** mortality, life span, athlete, health, achievement age, sport

## Abstract

**Objectives:**

To examine the precocity-longevity (P-L) effect in North American professional basketball players who debuted between 1946 and 1979, and to determine whether playing position and decade of play influenced the relationship between age of career achievements and life span.

**Methods:**

A total of 1852 players were evaluated from a recognized sports archive (i.e., http://sports-reference.com), which provided information on date of birth, death, and career debut, playing position, and indicators of achievement (i.e., All-Star team and/or All-League team selection). Athletes were categorized as above or below the median age of professional debut and median age of selection to first All-Star team and/or All-League team. Analyses of deceased players (*n* = 598) were comprised of bivariate correlations between age of achievement (age of debut, age of first All-Star game, and age of first All-League team selection) and age of death, and *t*-tests to compare the average age of death of early and late achievers (*p* < 0.05). Survival analyses, using the entire sample (living and deceased players), compared the life spans between those who debuted above and below the median age of achievement for each indicator of achievement.

**Results:**

Only the correlation between age of professional debut and age of death (*r* = 0.33, *p* < 0.001), age of first All-Star game and age of death (*r* = 0.29, *p* < 0.05), and the *t*-test comparing the average death age of early (66.4 years) and later (69.3 years) debut age groups (*p* = 0.01) reached statistical significance. However, survival analyses demonstrated a trend for lower risk of death for early achievers, with one exception (i.e., age of debut); this trend was not statistically significant.

**Conclusion:**

Results did not support the P-L hypothesis, suggesting that sample characteristics (i.e., physical fitness of high performance athletes), and measurement methodologies, may influence support for the proposed hypothesis in sport. However, future research would benefit form larger sample sizes and cause of death data.

## Introduction

The precocity-longevity (P-L) hypothesis asserts that those who experience noteworthy high achievements earlier in life have a shorter life span than those who attain similar notable career milestones later in life. This hypothesis was first formally described by McCann (1) in a study of high achievers in politics (presidents and prime ministers), law, entertainment, creative pursuits, and academia. Results suggested that the younger a person is when they achieve a notable peak in achievement (or a landmark contribution) the younger they tend to die. For example, Nobel Peace prize recipients who received the award above the median age of 64.8 years lived 13.5 years longer than those who received the prize before this age [*p* < 0.01; Ref. (1)].

Two mechanisms have been at the forefront of discussion (and have existed from the hypothesis’ inception): stress and personality type. First, McCann (1) suggested that the stress, challenges, and obligations that accompany precocious achievement have the potential to be a catalyst for poor health. McCann (1) further highlighted that the *ascension* to career success may play an important role: “the stresses that accompany a rapid drive to achievement peaks may eventually contribute to a shorter life” (p. 1430). Research does support that stressful experiences predispose humans to disease, and ultimately premature death [e.g., Taylor (2)], and that repetitive stress may activate physiological responses that cause stress-related diseases to emerge (3).

Personality type, the more stable and ingrained form of individual character, is the other proposed mechanism of the P-L effect (1). Type A personalities tend to be competitive, self-critical, ambitious, goal-oriented, impatient, and aggressive; alternatively, Type B personalities are relaxed and non-competitive (4). The seminal study by cardiologists Friedman and Rosenman (5) found that Type A personalities have a higher risk of heart disease and high blood pressure relative to Type B personalities. However, contemporary research suggests a more complicated relationship between components of Type A personality (i.e., hostility and anger) and disease [e.g., Myrtek (6)]. Nevertheless, previous studies on the P-L relationship may have examined samples that likely fall in some components of the Type A personality category, such as professional baseball players (7) and eminent persons such as presidents, prime ministers, and monarchs (1), making personality type a potential mechanism.

Although etiological studies of the P-L effect will be important, it has been suggested that a more immediate objective should be to determine the existence and generalizability of the phenomenon (8). To date, however, only a few studies have investigated the existence and generalizability of the P-L effect in the athletic disciplines. Abel and Kruger’s (7) investigation of deceased Major League Baseball (MLB) players noted that when precocity was defined as age in which athletes debut professionally, there was a significant relationship with life span longevity; for every year a player debuted before the average age of 23.6 years (SD = 2.3), their life span decreased by 0.24 years. Further, correlations between debut age and longevity were higher in Hall of Fame inductees relative to non-inductees (0.48 vs. 0.09, respectively), which further supported the proposition that precocious achievement may have negative effects on longevity. Alternatively, a recent analysis by Lemez et al. (9) on Canadian professional ice hockey players did not demonstrate a clear P-L effect. The authors speculated that the positive relationship between cardiovascular fitness and reduced all-cause mortality may explain the lack of an effect among ice hockey players compared to baseball and non-athletic eminent achievers. However, with only two studies to date on high achieving athlete populations, there is a clear need to explore the P-L effect in other athlete samples.

In addition to there being a limited number of studies on high performing athletic populations, there are also methodological challenges inherent to research on the P-L effect. First, there are some epidemiological and methodological influences that may affect the P-L effect: life expectancy and selection artifacts. The life expectancy artifact describes the potential that younger death age among high achievers may result from the fact that younger age is associated with shorter life expectancy (1). There is also the possibility that early death (and hence early achievement) simply permits a person to enter a study sample. This “selection artifact” (10) means that the absence of still-living early achievers may skew effects in favor of a P-L effect. Both of these artifacts describe the inherent potential for bias when studying a sample of *only* deceased eminent achievers and may increase the likelihood of Type I error (i.e., identifying a P-L effect when one does not exist).

There may also be challenges to studying the P-L effect as a result of how “precocity of achievement” is operationalized. Both Abel and Kruger (7) and Lemez et al. (9) defined precocious achievement in athlete samples as the age at which athletes entered professional sport. However, using age of professional sport debut as a measure of achievement may be impacted by the fact that the age range for professional sport debut is typically quite narrow (7). If this range is sufficiently narrow, it would be difficult to find clear support for the P-L effect as the age difference between “early achiever” and “late achiever” would be quite small. Lemez et al. (9) suggested that one way to circumvent these issues would be to use an alternate marker of accomplishment more independent of age and more indicative of exceptional achievement, such as age at first participation in an “All-Star” game.

Taken together, there is a need to study the generalizability of the P-L effect in different domains (e.g., sport vs. non-sport), and there is a need to start reconciling the methodological challenges inherent to studying this phenomenon. Based on the two studies of the P-L effect among eminent athletes, this study has two aims: (1) to explore the generalizability of the P-L effect among high achievers in a sample of athletes not previously studied (i.e., North American professional basketball players) and (2) to address some of the methodological challenges that are important to consider in this area of research. To that end, the current study aimed to explore the P-L effect using diverse approaches. First, we replicated previous methodologies for analyzing the effect using a sample of only deceased athletes in order to compare this sample to previous research. Second, the unique approach of analyzing an inclusive sample of deceased *and* living athletes allowed more ecologically valid analyses to be performed. Based on previous P-L research on athlete and non-athlete samples, we hypothesized that athletes who had younger ages of notable achievement would have shorter life spans than those who had notable achievement at older ages.

## Materials and Methods

### Sample and Variables

This study did not involve any human participants or animals and therefore adhered to recognized ethical standards and national/international laws (11). Data were collected for *all* players who had played professional basketball from 1946 to 2012 (*N* = 4015) from a recognized sports archive of aggregated publically available athlete records (i.e., http://sports-reference.com). During this period two professional leagues existed, the National Basketball Association (NBA) and the American Basketball Association (ABA). A random sample of the data (10%) was also cross referenced with an official NBA encyclopedia (12) and showed complete consistency. We censored all players who debuted from 1980 to 2012 because the majority (98.4%) of that cohort was still living. This resulted in a final sample size of 1852. Of this overall sample, 598 were deceased.

For each player, date of birth, career milestones (i.e., first year of professional debut, last year of play, date of first All-Star game, date of first All-League selection), playing position (i.e., Guard, Center, or Forward), and date of death were collected. The All-Star game is an annual mid-season exhibition game where 12 players from each conference (i.e., Eastern and Western) who are playing at a notably high-level are selected by fans (starters) and coaches (reserves). Similarly, an All-League selection honors the best players *following* a season to two teams from 1946 to 1988 (*N* = 10), which are selected by a panel of sportswriters and broadcasters throughout Canada and the United States [e.g., Ref. (13)]. From the above data, a number of additional variables were derived for the current study, including (i) length of playing career (in seasons played), (ii) age at the start of playing career, (iii) age at first All-Star game, (iv) age at first All-League team selection, (v) age at time of death (in years), and (vi) age as of January 2013 (days) for living and former athletes. Although age-related variables were computed and analyzed in “days,” results are presented in years for ease of understanding. Athletes were also coded as either deceased or living for the purpose of analyses.

Precocity of achievement was defined in three different ways. First, we replicated Abel and Kruger’s (7) definition of achievement as “age upon entering professional sport.” In addition, in order to have a measure of achievement more independent of age, and more indicative of eminent achievement in this population, two other indicators of achievement were included, “age at first All-Star game” and “age at first All-League selection.” For each indicator of achievement, variables were computed using a player’s *first* notable achievement. This was necessary because the ABA only existed from 1967 to 1976, and therefore some players debuted in the ABA and transferred to the NBA (and *vice versa*). For example, if a player debuted or was nominated to an All-Star game in the ABA then later transferred to the NBA, the achievement values were derived from their ABA. Consistent with previous research, we explored career precocity by coding athletes as debuting either below or above the median “age of achievement” for each indicator of achievement. The median ages of achievement were calculated separately for each decade of debut, since these values fluctuate (this was particularly important for debut age, which has declined in more recent decades).

### Analyses – Part 1 (Deceased Sample)

The purpose of our first set of analyses was to replicate the methods of previous studies in an unstudied population (i.e., professional basketball players) and with additional indicators of achievement. Specifically, these analyses used a subsample of *only deceased* players who had debuted prior to 1980 (*n* = 598), similar to previous research on eminent athlete and non-athlete samples (1, 7). As such, all coding of the achievement measures in this portion of the analyses were based on the values (i.e., median ages of achievement) obtained from the deceased subsample of NBA players.

We described the study sample by calculating the median age of death, the sample composition according to playing position (Center, Forward, Guard), and the proportion of deceased athletes who had participated in an All-Star game, and who had been selected to an All-League team.

In accordance with previous research [see McCann (1)], each of the three measures of achievement were correlated, and partially correlated (controlling for decade of debut), with age at death (*p* < 0.05). Similarly, *t*-tests compared the mean ages of death for those who were above and below the median ages of achievement for each of the three measures of achievement.

Two final analyses were included in Part 1. We also categorized athletes as above (later death) or below (early death) the median age of death (where each median death age was decade specific) and categorized athletes as either “eminent” or “not eminent” based on whether or not they had been selected as an All-Star or to an All-League team (respectively). Using these categorizations, two 2 × 2 chi-squares were run with a *p* < 0.05 significance criteria: All-Star eminence (yes/no) × death age (early/later); All-League team eminence (yes/no) × death age (early/later). Similar to Abel and Kruger’s (7) distinction between Hall of Fame and non-Hall of Fame athletes, these analyses sought to distinguish any potential differences in longevity associated with *degree* of eminence.

### Analyses – Part 2 (Alive or Deceased Sample)

The second set of analyses for this study aimed to address the possibility that a P-L effect observed in a sample comprised of deceased eminent achievers may result from still-living eminent achievers not having entered the study sample [i.e., the selection artifact: Simonton (10)]. Therefore, these analyses used a sample of deceased and still-living players who had debuted prior to 1980 (*n* = 1852) with the aim of testing whether or not those with earlier death age are in fact more precocious achievers than still-living athletes. As with the first portion of analyses, achievement was defined as (i) age of professional debut, (ii) age at first All-Star game, and (iii) age at first All-League team selection. However, when coding athletes as above or below the median age of achievement (for each indicator of achievement), the values of the *entire* sample (living and deceased) of athletes were used for these analyses (with median achievement values calculated separately for each decade of play and pertinent league).

We described the study sample by providing the proportions of each playing position (Center, Forward, Guard), and the proportion of the sample who had participated in an All-Star game, and who had been selected to an All-League team. We also describe the sample by listing the median age (in days) of achievement for the three indicators of achievement (stratified by decade) separately for deceased and still-living players.

Three Kaplan–Meier (KM) analyses were used to explore the univariate patterns and median survival between each career achievement (i.e., precocity) indicator and longevity (*p* < 0.05), and to show censoring. Cox proportional hazards regression was performed to assess the influence of career precocity on life span, while adjusting for potential confounders (i.e., playing position and decade of career debut). Hazard ratios (HR) and 95% confidence intervals (95% CI) were used to estimate the relationship between precocity and longevity, after adjusting for covariates.

## Results – Part 1 (Deceased Sample)

The median age of death among the deceased players was 70.3 years (SD ± 14.4 years). Overall, the majority of this sample was comprised of Guards (42.4%) and Forwards (42.5%), while Centers constituted only 15% of the sample. The majority of athletes participated exclusively in the NBA (87.9%), while smaller proportions played exclusively in the ABA (7.4%) or played in both leagues (4.7%). Of the 598 deceased athletes, 11.5% had participated in an All-Star game, and 6.3% had been selected to an All-League team.

Table 1 presents the correlations between each of the achievement measures and age of death, as well as the results of the independent *t*-tests. Both age of professional debut and age of first All-Star game demonstrated statistically significant positive correlations with age of death. The relationship between age of professional debut and age of death was a medium size correlation (*r* = 0.33) but decreased to a small correlation (*r* = 0.11) when decade of professional debut was adjusted for. The correlation between age of first All-Star game and age of death was also medium-sized (*r* = 0.29), but this correlation was not statistically significant when decade of debut was adjusted for. Age at first All-League selection was not significantly correlated with age of death (regardless of whether decade of debut was adjusted for). *T*-tests were used to compare the average age of death between earlier and later achievers (see Table 2). Although there was a trend for early achievers to have a younger mean age of death for each of the indicators of achievement, the difference in death age was only statistically significant between those with earlier and later ages of professional debut (*p* = 0.01).

**Table 1:** **Bivariate and partial correlations between age of achievement measures and age of death**.

Test correlations	Measure of achievement (age)	Death age (*r*)	*p* value	Death age (*r*)^a^	*p* value
	Age pro debut (*n* = 598)	0.33	<0.001	0.11	<0.01
	Age first All-Star game nomination (*n* = 69)	0.29	<0.05	0.22	0.07
	Age first All-League team nomination (*n* = 38)	0.10	0.54	0.27	0.10

*^a^Partial correlation controlling for decade of entry to professional basketball. All of the above analyses were repeated using restricted inclusion criteria that ensured no athlete had died before all the individuals in the sample had accomplished their measure of career achievement*.

**Table 2:** ***T*-test comparisons of the mean death age between earlier and later achievers**.

Test	Measure of achievement	Mean death age (years)		*t* (df)	*p* value
*T*-tests	Above vs. below median achievement age^a^	Earlier achiever M (SD)	Later achiever M (SD)		
	Age of pro debut (*n* = 598)	M = 66.4 (±15.2)	M = 69.3 (±14.3)	*t* (596) = 2.34	0.02
	Age first All-Star game nomination (*n* = 69)	M = 62.2 (±16.7)	M = 67.7 (±9.6)	*t* (67) = 1.65	0.10
	Age first All-League team nomination (*n* = 38)	M = 63.1 (±17.4)	M = 69.1 (±10.1)	*t* (36) = 1.27	0.21

*^a^Age of median achievement was calculated for each decade, and median splits were respective to athletes’ decade of entry to professional sport; M, mean. All of the above analyses were repeated using restricted inclusion criteria that ensured no athlete had died before all the individuals in the sample had accomplished their measure of career achievement*.

Neither the chi-square test for All-Star eminence (yes/no) × death age [above vs. below median; χ^2^(1) = 0.13, *p* = 0.72] nor the chi-square test for All-League team eminence (yes/no) × death age [above vs. below median; χ^2^(1) = 1.07, *p* = 0.30] provided any evidence for the P-L hypothesis.

## Results – Part 2 (Alive or Deceased Sample)

**Figure 1:**
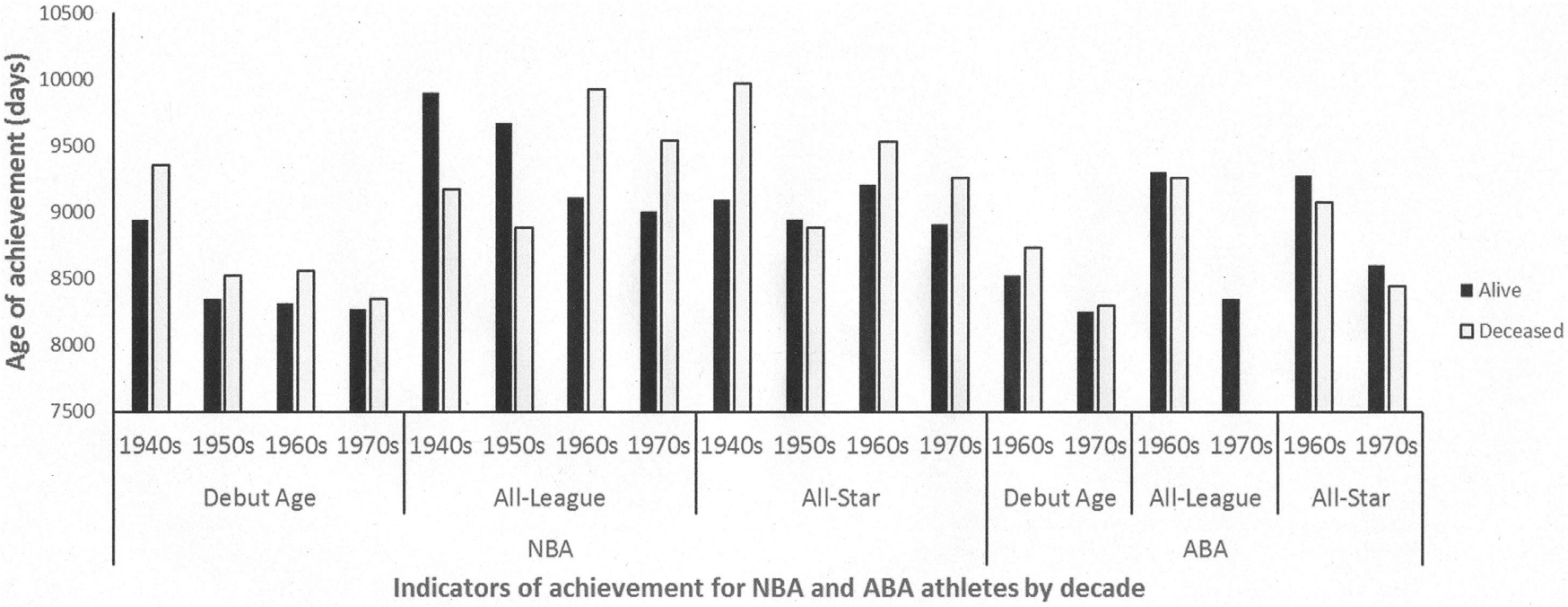
**Median age of achievement for alive and deceased NBA/ABA athletes, for debut age, All-League nomination, and All-Star nomination**.

Of the 1852 players alive or deceased, the majority of the sample was comprised of Guards (42.4%) and Forwards (42.5%), while Centers constituted 15.1% of the sample. The majority of athletes participated exclusively in the NBA (73.4%), while smaller proportions played exclusively in the ABA (15.5%) or played in both leagues (11.1%). Overall, 14.7% had participated in an All-Star game, and 6.7% had been selected to an All-League team. Descriptive comparisons of median age of achievement between living and deceased players revealed that living athletes had younger median ages of achievement on all *Age of Professional Debut* comparisons, and 5 out of 11 comparisons for *Age at first All-Star* and *All-League team selection* (see Figure 1).

### Age of Professional Debut

The median life span for early achievers was higher than it was for late achievers (82.6 vs. 81.0 years), although this trend was not statistically significant [χ^2^(1, *N* = 1852) = 3.31, *p* = 0.07]. In a subsequent Cox regression, which adjusted for playing position and decade of playing debut (see Table 3), career precocity was associated with a lower risk of death (HR: 0.84, 95% CI: 0.71–0.98). No main effects were observed for decade of debut. However, compared to Guards, Forwards had a higher risk of death (HR: 1.21, 95% CI: 1.02–1.45), but no difference in risk of death among Centers. No two-way interactions were observed.

**Table 3:** **Hazard ratios for age of achievement, decade of debut, and playing position from Cox regressions for age of debut, age of first All-Star game, and age of first All-League team indicators of achievement**.

			Mortality risk	
Measure of achievement	Variables		HR_adjusted_	95% CI
Age of debut	Age of achievement	Early achievement	**0.84**	**0.71–0.98**
		Late achievement	**–**	–
	Decade of debut	1940s	1.01	0.81–1.51
		1950s	0.95	0.69–1.33
		1960s	0.87	0.63–1.20
		1970s	–	–
	Playing position	Centers	1.20	0.94–1.52
		Forwards	**1.21**	**1.02–1.45**
		Guards	–	–
Age at first All-Star game	Age of achievement	Early achievement	0.82	0.54–1.44
		Late achievement	–	–
	Decade of debut	1940s	0.96	0.36–2.55
		1950s	1.14	0.45–2.85
		1960s	0.81	0.34–1.91
		1970s	–	–
	Playing position	Centers	1.40	0.71–2.75
		Forwards	1.64	0.95–2.83
		Guards	–	–
Age at first All-League team	Age of achievement	Early achievement	0.80	0.40–1.61
		Late achievement	–	–
	Decade of debut	1940s	0.68	0.21–2.19
		1950s	0.57	0.18–1.87
		1960s	0.55	0.17–1.74
		1970s	–	–
	Playing position	Centers	1.29	0.50–3.34
		Forwards	1.38	0.66–2.88
		Guards	–	–

*HR, hazard ratio; CI, confidence interval; bold font, statistically significant HR*.

### Age at First All-Star Game

Although the median life span for early achievers was higher than it was for late achievers (84.1 vs. 80.7 years) in the current sample, this trend was not statistically significant [χ^2^(1, *N* = 271) = 0.05, *p* = 0.83]. In a subsequent Cox regression (see Table 3), which adjusted for playing position and decade of playing debut, career precocity was not independently associated with a lower risk of death. Moreover, no main effects were observed for decade of debut or playing position, and no interactions were observed.

### Age at First All-League Team Selection

The median life span for early achievers was lower than it was for later achievers (82.4 vs. 84.1 years), although this trend was not statistically significant [χ^2^(1, *N* = 119) = 0.10, *p* = 0.75]. When decade of debut and playing position were adjusted for in a Cox regression (see Table 3), mortality risk did not differ between early and later achievers. No main effects were observed for decade of debut or playing position, and no interactions were observed.

## Discussion

This study examined the relationship between precocity of achievement and longevity in elite professional basketball players. Previous work by McCann (1) and Abel and Kruger (7) observed that earlier achievement is associated with a shorter life span across different domains, including sport. However, the current results present a complicated picture of the P-L effect. Our interpretation is that overall the results do not support the P-L hypothesis.

First, the only statistically significant correlation and *t*-test were for the age of debut indicator of achievement. None of the Part 1 analyses on the age at first All-Star game and age at first All-League selection reached statistical significance, although that may have resulted from lack of statistical power. The results of the chi-square tests for these two indicators of achievement also did not support the P-L hypothesis. However, given the small sample sizes in these analyses, there is the need to weigh the results of the first series of analyses against the results of Part 2.

The second series of analyses aimed to expand on the methods used in the majority of P-L research. It had previously been suggested that younger death age among high achievers results from lower life expectancies associated with younger age (2) and that early death may simply permit a person to enter a study sample [i.e., the selection artifact: Simonton (10)]. The inclusion of still living and deceased athletes and the subsequent results of the survival analyses (Part 2) suggest that the concerns related to the above artifacts are well founded in the current population of high achievers. Not only did the results of the survival analyses not support the P-L hypothesis, the trends for the three indicators of eminent achievement were opposite to those in Part 1 and suggest a *lower* risk of death for precocious achievers. Although only the age of debut achievement indicator reached statistical significance, the other two indicators of achievement has similar effect sizes and may have been significant with larger sample sizes. There is of course a notable limitation to using survival analyses in instances where the exposure event (age of achievement) is itself a potential confounder of the outcome, which cannot be controlled for. In this case, it is not possible to control for the fact that precocious achievers are followed for a greater period of time than late achievers. The results of the current study should be weighed against such limitations [see Hanley and Foster (14)]. However, in light of the notable limitations to the methods presented in Part 1, we argue that survival analyses perhaps represents the lesser of two evils, and that for the time being a triangulation of multiple methods, which includes deceased-only and still-living athletes, may be prudent. It may be worth repeating; however, that one of the biases identified earlier is that precocious achievement and younger age is associated with greater risk of mortality (1, 2). As such, if the limitations inherent to survival analyses biased our findings, they theoretically should have reflected biased support *for* the P-L hypothesis. However, the results of the survival analyses do not conclusively support the P-L effect. We believe that these trends stem from the differences in age of achievement between living and deceased athletes.

The discrepancy between the trends observed in Part 1 and Part 2 appear to be related to the different median age of achievements between still-living and deceased athletes (Figure 1). Descriptive trends suggest that in many cases the median achievement age of *deceased* athletes does not accurately reflect the median achievement age for the overall population of athletes. In some cases, it appears that the median age of achievement of the still-living athletes is actually younger than that of the deceased-only subsample, especially with regard to the “age of debut” measure of achievement. These descriptive differences in median age of achievement suggest there is a P-L effect in the current sample, but unlike previous research, precocity of achievement appears to be related to *longer* life span in this population. The fact that the median age of achievement differs for deceased and still-living athletes in many cohorts (i.e., decade of entry and league) of athletes suggests that this trend may be systematic, not random, and that the Part 2 analyses are a more accurate reflection of achievement and mortality trends within this population. It also introduces the possibility of an additional bias; that the median age of achievement in previous studies of deceased-only samples may not accurately reflect the true median value for the entire population of eminent achievers. As Hanley and Foster (14), p. 8 suggest, “Theories such as the just-cited precocity-longevity hypothesis are seductive, and have a certain plausibility. But some of this may be a result of the framing.” This seems to be the case for our data; as such, we conclude that the trends observed in Part 1 appear to be less representative of achievement trends than those observed in Part 2.

There are many reasons that the P-L effect may not exist in the current sample of high achievers. As Lemez et al. (9) noted, there are notable differences between previous research and the current investigation that might explain these discrepancies. First, given the importance of physical fitness for the reduction of all-cause mortality (15), it is possible that the high levels of physical fitness required for elite performance in basketball players compared with the domains examined by McCann (1) and the baseball players examined by Abel and Kruger (7) buffered the effect of precocity on longevity. Moreover, beginning a professional career earlier and having the potential for longer exposure to financial rewards, health care, and fitness-related health benefits may explain the finding that early achievers have longer life spans in this sample. A related explanation is that players who eventually make it to play professional basketball, regardless of their age of debut, have a superior genotype for physical fitness than those in other domains, which negates any relationship between early achievement and length of life span. The “Healthy Worker Effect” describes a form of selection bias wherein inclusion into a study sample reflects a health-advantage compared to those not included [see Rothman (16)]. As such, the Part 1 analyses of deceased-only athletes, which appear to support the trend for the P-L hypothesis, may reflect a sort of “inverse-healthy worker effect.”

Although the results of the current study add to a relatively limited evidence base regarding predictors of mortality in elite and professional sport, the current study did not provide any insights on cause-specific mortality. Nor can it be said at this point that the P-L hypothesis can be conclusively rejected. Future investigations would benefit from larger sample sizes, and from more nuanced categorizations of “age of achievement” (e.g., a three or four level categorical age of achievement variable rather than a dichotomized median split). It may also be useful to incorporate cause-specific mortality, and to explore disease links to sport participation, as well as any subsequent links to the proposed underlying mechanisms of the P-L effect, such as stress and personality. These areas may provide further insight into the long-term risks and benefits of participation in elite sport. The historical nature of this sample, and the possibility of generational differences in mortality trends and life expectancy, also reinforce the need to incorporate cause-specific mortality in future research. It will be important to consider these factors when exploring the generalizability of the current findings in other samples of eminent athletes.

In summary, the results of the current study suggest it is unlikely that the P-L effect exists in NBA athletes. Importantly, the current study also raises a methodological consideration regarding the estimates of average precocity values, and whether or not the average age of precocious achievement among deceased eminent achievers is an accurate reflection of the entire eminent population. An average age of achievement among still-living eminent achievers that is significantly different than that of deceased eminent achievers could skew the results (as was likely the case for our data). Going forward, it will be important to consider how the physical demands of a sport, the health and financial benefits-associated with participation in high performance sport, and measurement methodologies may influence support for the P-L hypothesis in sport. Given the significant dedication toward talent identification and development at early ages in many sports worldwide, the potential relationship between the milestones of athlete development and athlete health outcomes are important to consider.

## Author Contributions

The authors NW, SL, CA, MR, and JB contributed equally to this work.

## Conflict of Interest Statement

The authors declare that the research was conducted in the absence of any commercial or financial relationships that could be construed as a potential conflict of interest.
